# Empirically-Derived Dietary Patterns in Relation to Non-Alcoholic Fatty Liver Diseases Among Adult Participants in Amol, Northern Iran: A Structural Equation Modeling Approach

**DOI:** 10.3389/fnut.2022.821544

**Published:** 2022-03-28

**Authors:** Azam Doustmohammadian, Elham Pishgar, Cain C. T. Clark, Elham Sobhrakhshankhah, Mehdi Nikkhah, Amir Hossein Faraji, Nima Motamed, Mohsen Reza Mansourian, Bahareh Amirkalali, Mansooreh Maadi, Maryam Sadat Kasaii, Hamidreza Ebrahimi, Farhad Zamani

**Affiliations:** ^1^Gastrointestinal and Liver Diseases Research Center, Iran University of Medical Sciences, Tehran, Iran; ^2^Centre for Intelligent Healthcare, Coventry University, Coventry, United Kingdom; ^3^Department of Social Medicine, Zanjan University of Medical Sciences, Zanjan, Iran; ^4^National Nutrition and Food Technology Research Institute, Shahid Beheshti University of Medical Sciences, Tehran, Iran

**Keywords:** dietary pattern, non-alcoholic fatty liver disease, NAFLD, structural equation modeling, adult

## Abstract

Dietary modifications remain the mainstay in managing nonalcoholic fatty liver disease (NAFLD). Published data on the effect of overall dietary patterns on NAFLD is scarce. The present study aims to extract the dietary patterns and investigate their association to NAFLD by gender, using structural equation modeling, among adult participants in Amol, northern Iran. In this cross-sectional study, data from 3,149 participants in the Amol cohort study (55.3% men, *n* = 1,741) were analyzed. Usual dietary intake was assessed by a validated 168-items semiquantitative food frequency questionnaire. We classified major dietary patterns by explanatory factor analysis (EFA) and confirmatory factor analysis (CFA). NAFLD diagnosis was based on ultrasound scanning, including increased hepatic echogenicity, abnormal appearance of hepatic arteries, and diaphragm in the absence of excessive alcohol consumption. Multivariable logistic regression and structural equation modeling (SEM) were used to explore the relationship between dietary patterns and NAFLD. Three distinct dietary patterns, including western, healthy, and traditional/mixed dietary patterns, were identified. Adult male who adhere to the western dietary pattern were more affected with NAFLD risk [Q1, Q2, Q3, Q4, odds ratio *(OR)* = 1, 1.16, 1.34, 1.39; 95% confidence interval *(CI)* = 0.83–1.61, 0.96–1.85, 0.98–1.96, *p*_*trend*_ = 0.04, respectively]. A full mediating effect of healthy dietary pattern, western dietary pattern, and traditional dietary pattern *via* dietary acid load (DAL) proxy (of dietary patterns to DAL: βstd = −0.35, *p* < 0.006, β*std* = 0.15, *p* = 0.009, and β*std* = 0.08, *p* = 0.001, respectively), on NAFLD was found through mediation analysis using SEM. A western dietary pattern comprising frequent intake of salty and sweet snacks, soft drinks, refined grains, processed meats, cooked and fried potatoes, eggs, and coffee was associated with a higher odds of NAFLD in an Iranian male population. Additionally, our findings might provide a mechanistic explanation for the association between dietary patterns and NAFLD *via* DAL proxy. However, further prospective studies, including assessing acid-base biomarkers, are needed.

## Introduction

Non-alcoholic fatty liver disease (NAFLD) is the most common liver disorder globally and is projected to become the leading cause of cirrhosis, hepatocellular carcinoma, and liver transplant over the ensuing decades (1). The global prevalence of NAFLD is estimated at 25%, rising inexorably because of significant changes in lifestyle in recent decades (1). Indeed, a meta-analysis of 237 studies in Asia with a total sample size of 13 million adults found that the overall prevalence of NAFLD was around 29.62%, regardless of the diagnostic method (2). Moreover, contemporary evidence indicates a rapid increase in a load of this disease in Iran, where, according to a meta-analysis study, the prevalence of NAFLD was 33.9% (3).

Although NAFLD is a globally prevalent and rapidly growing epidemic, there is no universally approved pharmacological treatment (4). NAFLD is caused by the interaction between genetics, environment, and human behaviors (5); however, several studies have posited that diet as an important pathogenic factor of NAFLD (6, 7). Available evidence suggests that the only effective treatment is a change in lifestyle and dietary pattern (8). Compared to traditional dietary analysis, the analysis of dietary patterns has been considered as a comprehensive approach to better reflect the potential synergistic effects of foods and nutrients of complex diets rather than focused on individual nutrients or foods (6, 9). Additionally, dietary patterns are more consistent across time and significantly affect health outcomes to a greater extent than individual nutrients (10).

On the basis of extant literature, obesity, type 2 diabetes, and metabolic syndrome are significant risk factors for developing NAFLD (5), and unhealthy dietary patterns may be associated with NAFLD risk considering their relationship with obesity and other risk factors for degenerative disease (11).

Previous studies have revealed that adherence to a western dietary pattern may increase the risk of NAFLD, whereas prudent and Mediterranean dietary patterns are more likely to be associated with a reduced risk of this disease (7, 12–14). To our knowledge, no existing studies have explained the direct and indirect associations among different dietary patterns and NAFLD on a large scale. Structural equation modeling (SEM) is considered a suitable statistical method to test the validity of dietary patterns and identify the direct and indirect relationship between potential and observation variables through combining factor analysis and path analysis (15, 16). SEM incorporates simultaneous structural equations where variables may influence each other reciprocally, directly or indirectly, through other variables as mediators. This approach provides a more in-depth insight into the assessment of NAFLD and its determinants; individual differences and errors are also considered in SEM (17). Therefore, the aim of this study is to use SEM to extract the dietary patterns among Iranian adults in Amol, northern Iran, and investigate their association with NAFLD by gender.

## Methods

### Study Design and Population

In this cross-sectional study, data were derived from the Amol cohort study (second phase in 2016), consisting of 6140 individuals aged 10–90 years. The sampling frame was obtained from twenty-five rural and sixteen urban local health centers in Amol, northern Iran. The city population was then classified into 16 strata based on gender and age at 10-year intervals. The sampling strategy is described in detail elsewhere (18).

The study subjects were adults aged ≥18 years, of Iranian nationality, willing to participate in the study, and lifelong residents in Amol city. In collaboration with health care centers, demographic, biochemical, anthropometric, medical, dietary, and physical activity data (documented in 2016) were collected through a face-to-face interview conducted by an expert research team.

On the basis of the study design, 5,147 adults aged ≥18 years (18–90) were included. In the second phase of the cohort study, data on 459 individuals were unavailable due to death (*n* = 77), non-cooperation (*n* = 357), or migration (*n* = 25). Individuals following a specific dietary or physical activity regimen (due to a particular disease, losing weight, or professional exercise), history of hepatic diseases, such as Wilson's disease, autoimmune liver disease, hemochromatosis, virus infection, and alcoholic fatty liver, malignancy, thyroid disorder, and autoimmune diseases, as well as participants with significant alcohol consumption (>30 g/d for men and >20 g/d for women) (19, 20) (*n* = 486), and lactating/pregnant women (*n* = 153), were excluded from the study. Participants with missing data, such as abdominal ultrasonography (*n* = 166), covariates (*n* = 186), food frequency questionnaire (*n* = 249), and individuals with extreme energy intake values (according to the criteria of Willett (21) defined as <800 and >4,000 kcal/day for men and <600 and >3,500 kcal/day for women) (*n* = 299), were also excluded. Finally, 3,149 subjects, consisting of 1,408 women and 1,741 men, were eligible for analysis.

The sociodemographic profile of those who were excluded from the study didnot differ significantly from that of the remaining participants. The Iran University of Medical Science (IUMS) ethical committee (No. IR.IUMS.REC.1400.162) funded and approved the study protocol, and all participants signed an informed consent form prior to the study commencement.

### Clinical Assessments

Blood pressure (mmHg) was measured according to the standard recommendations (22) using a sphygmomanometer cuff (Riester GmbH, Jungingen, Germany), while the participants rested for 15 min in a comfortable, seated position. Measurements were performed twice at 1 min intervals, and the mean of the two measurements was recorded as systolic and diastolic blood pressure.

Non-alcoholic fatty liver disease diagnosis was based on ultrasound scanning such as increased hepatic echogenicity, abnormal appearance of hepatic arteries and diaphragm in the absence of excessive alcohol consumption, drug-related steatosis, and viral or hereditary hepatic liver conditions (19, 20). Sagittal, longitudinal, lateral, and intercostal views of the liver parenchyma were analyzed in radiology using a low-frequency convex transducer (2–5 MHz).

Ultrasound examinations were performed by a radiologist who was completely blind to the study protocol and while the subjects were fasting.

### Laboratory Assessments

Following 12 h of fasting, a venous blood sample was drawn from each participant to determine the fasting blood sugar (FBS) and lipid profiles. According to the protocol using the BS200 Auto analyzer (Mindray, China), all tests such as FBS, triglyceride, high-density lipoprotein (HDL), and total cholesterol, were assessed enzymatically. Serum low-density lipoprotein (LDL) cholesterol was calculated using the Friedewald equation (23).

Acon kits (Acon Laboratory, San Diego, CA92121, USA) were used to evaluate hepatitis B virus (HBV) biomarkers such as HbsAg, HBsAb, and HBcAb using the third generation Enzyme-Linked Immuno-Sorbent Assay (ELISA) method.

The Iranian National Reference Laboratory re-evaluated 10% of the blood samples, and a variation coefficient of 1.7–3.8% was considered acceptable for all laboratory values.

### Anthropometric Assessment

Anthropometric measurements were performed with participants wearing light layer of clothing and no shoes. Weight (kg) and height (cm) was measured, and body mass index was calculated. Waist circumference was measured by a well-trained researcher using a non-stretch measuring tape to the nearest 1 mm. All measurements were carried out twice using standard protocols and techniques (24).

### Dietary Assessment

Nutritionists gathered all the information through face-to-face interviews. A validated Iranian questionnaire of 168 semiquantitative food frequency (FFQ) items was used to assess the usual diet (20, 23). Trained interviewers asked participants to report the frequency and amount of each food consumed during the past year on a daily, weekly, or monthly basis.

Food items included in the FFQ were grouped into 27 different categories according to similarities in nutrient contents and available data (19, 25–27) for factor analysis in our study. Some food items were considered separately because their nutrient content was unique (e.g., eggs, condiments, salt, tea, coffee). Table 1 indicates the components of food groups included in dietary patterns.

**Table 1:** Components of food groups included in dietary pattern.

**Foods or food groups**	**Food items**
Whole grains	Brown bread; Barely; Corn
Refined grains	White bread; Crispbreads; Spaghetti; Cooked rice; Low-sugar dry biscuits
Potatoes	Boiled potato; Cooked potato
Fried potatoes	Fried potatoes
Fast foods	Pizza
Processed meats	Sausage; kielbasa
Red meat	Beef meat & Lamb meat (tournedos, steak, mince); Hamburger
Organ meats	Organs meats of lamb and/or beef: liver, brain, tongue
White meats	Fish; poultry; canned tuna
Eggs	Eggs
Legumes	Lentils; Beans; Peas; Chickpeas; Soybeans
Nuts	Nuts and seeds
Vegetable oils	Olive oil; Sesame oil; Canola or Sunflower oil; Vegetable hydrogenated oil
Solid fats	Solid fat; Animal oils; Cream; Butter
Dairy products	Plain semi-skimmed milk; Plain skimmed milk; Low-fat yogurts; Low-fat yogurts, soft white cheeses; Lower-fat cheeses; Plain whole milk; Flavored milk; Natural whole yogurts; Soft rind cheeses
Dairy drinks	Dough; Buttermilk
Fruits	All types of fresh fruits, dried fruits, dates, fresh fruit juices
Tomatoes	Tomato; Tomato sauce
Vegetables	All types of fresh, cooked, and dried vegetables as well as vegetable juice
Salty & sweet snacks	Salty biscuits; Crisps; Crackers; Cookies; Brown or white sugar; Sweets; Cakes; Honey; Jam; Candy; Halva; Chocolates; Candied fruit
Soft drinks	Carbonated Sugar-sweetened soft drinks; Industrial fruit juices
Sauces	Mayonnaise
Pickles	Sour and salty pickles
Condiments	Condiments
Coffee	Coffee
Tea	Tea
Salt	Salt

In the present study, net acid excretion (NAE) was used to estimate dietary acid load (DAL). NAE was also estimated through the following formula:

NAE (mEq/day) = potential renal acid load (PRAL) + organic acids (OA) (28).

First, we calculated the PRAL using the suggested model by Remer as follows: PRAL (mEq/day) = [protein (g/d) × 0.49 + phosphorus (mg/d) × 0.037]–[potassium (mg/d) × 0.0211–magnesium (mg/d) × 0.0263–calcium (mg/d) × 0.013] (29). Then OA was estimated as OA (mEq/day) = [body surface area (m^2^) × 41 (mEq/day per 1.73 m^2^)/1.73 m^2^].

Body surface area was also calculated using the Du Bois formula (30, 31), as follows: Body Surface Area (m^2^) = 0.007184 × height (cm)^0.725^ × weight (kg)^0.425^.

In order to calculate dietary energy density (DED), total daily energy intake (kcal/d) was divided into total weight (g/d) of consumed food and caloric drinks (e.g., milk, dairy drinks, soft drinks, fruit juices). Non-caloric drinks, such as tea, coffee, and herbal drinks, were excluded from the DED calculation (32, 33).

### Preliminary Exploration of Dietary Patterns

Exploratory factor analysis (EFA) was applied to identify dietary patterns from 27 food groups. Sample adequacy by factor analysis was assessed using Kaiser-Meyer-Olkin (KMO) measure and Bartlett test of sphericity. Orthogonal varimax rotation was applied to decrease factor correlation and improve the interpretability of the factors. The values of KMO were 0.7 in men and 0.73 in women, respectively. Moreover, Bartlett's sphericity test was significant in both the genders (*p* < 0.001). The KMO and Bartlett test results showed that the diet data would be eligible for further analysis. The number of factors was determined using orthogonal rotation with the Kaiser criterion (eigenvalues >1.5) and scree plot. Scores for each extracted dietary pattern were calculated by aggregating the standardized intake of each food group associated with that pattern weighted by its respective factor loading. Food groups with factor loadings >0.20 (13, 34, 35) were included in the analysis, representing the foods that had the most robust relationship with the identified factor (see Table 2). Major dietary patterns were labeled according to the highest factor loading and interpretability. We also categorized the subjects into quartiles based on the distribution of factor scores in each stratum.

**Table 2:** Factor loadings for dietary patterns extracted from factor analysis by gender^a^.

**Food groups**	**Male**			**Female**		
	**Traditional** **pattern**	**Healthy** **pattern**	**Western** **pattern**	**Mixed** **pattern**	**Healthy** **pattern**	**Western** **pattern**
Liquid oils	**0.762**			**0.609**		−0.344
Organ meats	**0.743**			**0.743**		
Pickles	**0.546**	−0.214		**0.661**		
Solid fats	**0.424**			**0.213**		
Sauces	**0.411**		0.305	**0.415**		
Red meat	**0.377**			**0.453**		
Legumes	**0.319**			**0.353**		
Salt	**0.286**	0.201		**0.228**		
Tea				−0.257		
Vegetables		**0.780**			**0.762**	
Tomatoes		**0.651**			**0.660**	
Fruits		**0.517**			**0.431**	
Dairy drinks		**0.442**			**0.312**	
Dairy products		**0.425**			**0.352**	
Condiments		**0.353**			**0.376**	
White meats		**0.272**			**0.264**	
Nuts	0.268		**0.345**	**0.444**	0.258	
Processed meats			**0.401**	**0.358**		**0.235**
Salty & Sweet snacks			**0.546**	**0.570**		**0.354**
Soft drinks			**0.554**			**0.539**
Refined grains	−0.225		**0.452**			**0.533**
Eggs			**0.457**			**0.497**
Fast foods			**0.358**	0.341		**0.401**
Cooked potatoes		0.253	**0.325**			**0.350**
Fried potatoes			**0.300**			**0.423**
Coffee			**0.316**			**0.204**
Whole grains variance explained (%)	9.47	17.95	25.74	11.99	19.68	27.35

a *Values < 0.20 were excluded for simplicity*.

*Factor loadings ≥ 0.2 are in bold font*.

### Assessment of Other Variables

Subjects were asked to complete a questionnaire containing information about age (number of years according to their identification documents), gender (male or female), residency area (rural or urban), viral hepatitis (hepatitis C or hepatitis B), drug use history (steatogenic or hepatotoxic), and smoking (no smoking or current/past smoking).

Data on physical activity were obtained through a validated international physical activity questionnaire (IPAQ), which was presented as metabolic equivalent minutes per minute per week (MET-min/week) (36). A trained dietitian completed all the questionnaires.

### Statistical Analysis

Categorical variables were presented as a number or percentage, while continuous variables were presented as mean ± standard deviation (*SD*). A chi-squared test was used to compare the differences in the characteristics of the participants of categorical data.The *t*-tests were used to compare continuous variables.

We performed a multivariable-adjusted OR and 95% CIs to find the association between dietary patterns and NAFLD. In this regard, three models of logistic regression were assessed; model 1 was adjusted for demographic factor (age), model 2 was adjusted for demographic and lifestyle factors (age, smoking, waist circumference, physical activity, energy intake, lowering serum lipid drugs, lowering serum glucose drugs, and anti-hypertensive drugs), and model 3 was further adjusted for residual areas, family history of hypertension, CVDs, and diabetes.

**Figure 1:**
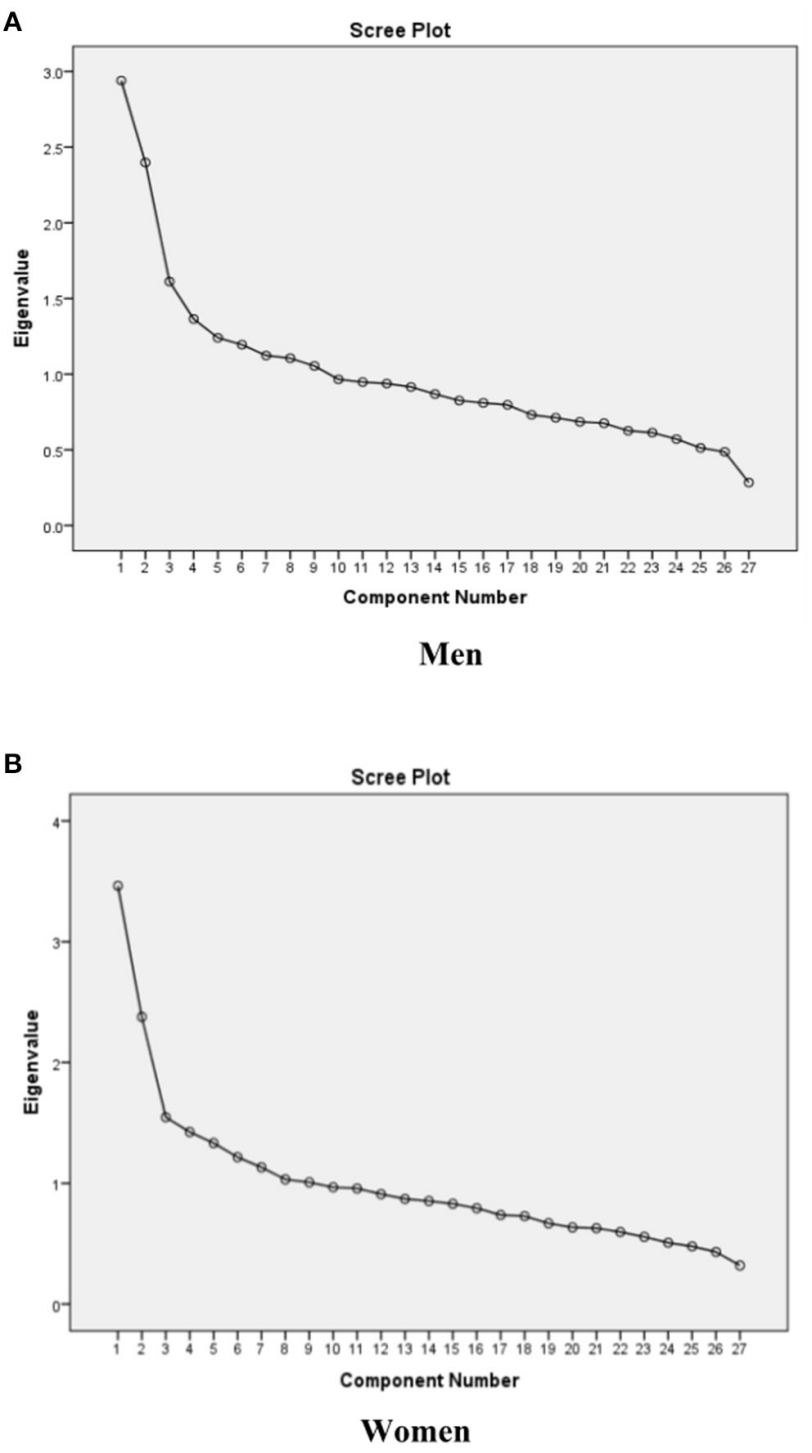
Scree plots of the eigenvalues to determine the appropriate number of dietary patterns among **(A)** men, **(B)** women.

Major dietary patterns were extracted using Principal Component Analysis (PCA) and orthogonally underwent varimax rotation (9, 13). The number of the extracted factors was chosen based on the eigenvalue factor (>1.5), scree plot (Figure 1), and factor interpretability (37). Confirmatory factor analysis (CFA) was performed to assess the relationship between observed variables and their underlying latent factors (here, the constructs of dietary patterns). The hypothesis of direct and indirect relationships between latent and residual variables was verified by SEM.

Goodness-of-fit to ensure that the proposed model can adequately explain the data was assessed by several models fit indices, including; χ^2^, the ratio of the χ^2^ to degrees of freedom (CMIN/DF), goodness of fit index (GFI), Adjusted GFI (AGFI), SRMR, and the Root Mean Square Error of Approximation (RMSEA). RMSEA and SRMR ≤ 0.08, and CMIN/DF < 4.0, are considered to represent an appropriate model fit to the data. For GFI, and AGFI, which range from 0 to 1.0, values >0.90 suggest an appropriate model fit the data (38).

We used IBM SPSS (version 22.0) and Amos (version 22.0) (IBM Corp, Armonk, NY, USA) for the statistical analysis (39). All *p*-values were two-sided, and statistical significance was defined as a *p* < 0.05.

## Results

### General Characteristics and Dietary Intake of Participants

According to the gender and the presence of NAFLD, the characteristics of participants were described in Table 3. Overall, 3,149 participants (55.3% male, *n* = 1,782) with complete data were analyzed in the study, out of which 1,437 (44.6%) subjects were diagnosed with NAFLD. The average of all laboratory tests, anthropometric measures, energy intake, and DAL proxy (NAE), as well as the frequency of disease history, the use of lowering serum lipid and glucose agents, and family history of hypertension were significantly different in between men and women (all *p* < 0.001). Men, compared to women, had a higher level of triglyceride (*p* = 0.002), energy intake (*p* < 0.001), CVDs (*p* = 0.005), and family history of diabetes. Among all participants, 25.8% (*n* = 450) of men engaged in smoking behavior, which was significantly higher than women (0.6%) (*p* < 0.001).

**Table 3:** Characteristics of the study participants: adults (*n* = 3,149, aged ≥18 years) at baseline, Amol Cohort Study, Iran, 2016–2017.

**Characteristic**	**Women** **1,438 (44.7)**	**Men** **1,782 (55.3)**		**Non-NAFLD** **1,783 (55.4)**	**NAFLD** **1,437 (44.6)**	
	**Mean (SD)**	**Mean (SD)**	***P*-value**	**Mean (SD)**	**Mean (SD)**	***P*-value**
Age (years)	45.80 (13.96)	48.22 (14.99)	<0.001	45.88 (16.05)	48.64 (12.46)	<0.001
BMI (kg/m^2^)	29.59 (5.33)	26.81 (4.35)	<0.001	25.96 (4.39)	30.54 (4.53)	<0.001
Energy intake (kcal/d)	2,166.95 (597.58)	2,455.83 (686.18)	<0.001	2,320.21 (668.11)	2,334.55 (658.55)	0.55
Waist circumference (cm)	87.07 (12.03)	89.72 (10.49)	<0.001	83.97 (9.96)	94.95 (9.64)	<0.001
Triglyceride (mg/dl)	128.46 (88.87)	138.42 (91.28)	0.002	111.59 (64.92)	160.63 (107.50)	<0.001
Total cholesterol (mg/dl)	183.66 (42.07)	178.36 (38.8)	<0.001	176.15 (39.29)	186.19 (41.08)	<0.001
HDL (mg/dl)	46.09 (11.80)	41.77 (11.40)	<0.001	44.72 (11.89)	42.48 (11.52)	<0.001
LDL (mg/dl)	99.71 (26.76)	98.76 (26.22)	0.31	97.45 (26.75)	101.26 (25.97)	<0.001
SBP (mmHg)	113.49 (20.44)	115.91 (18.11)	<0.001	111.20 (18.31)	119.15 (19.40)	<0.001
DBP (mmHg)	70.82 (12.28)	72.44 (11.32)	<0.001	69.14 (11.06)	74.78 (11.89)	<0.001
FBS (mg/dl)	108.71 (40.62)	103.92 (30.04)	<0.001	101.12 (31.21)	111.97 (38.70)	<0.001
ALT (mg/dl)	19.80 (13.96)	27.58 (20.32)	<0.001	20.15 (14.62)	28.81 (20.70)	<0.001
AST (mg/dl)	19.48 (8.26)	23.34 (11.44)	<0.001	20.72 (10.79)	22.69 (9.62)	<0.001
GGT (mg/dl)	24.15 (18.65)	29.53 (19.20)	<0.001	23.93 (18.01)	30.93 (19.74)	<0.001
ALKP (mg/dl)	195.73 (67.43)	199.45 (53.23)	0.09	192.87 (62.05)	203.65 (56.96)	<0.001
DAL (NAE)	1.47 (0.41)	1.56 (0.52)	<0.001	38.21 (23.35)	42.27 (25.13)	<0.001
DED	1.47 (0.41)	1.56 (0.52)	<0.001	1.52 (0.49)	1.52 (0.46)	0.45
	***N*** **(%)**	***N*** **(%)**	***N*** **(%)**	***N*** **(%)**	***N*** **(%)**	***P*** **-value**
Current/past smoker (%)	8 (0.60)	450 (25.80)	<0.001	276 (16.10)	182 (12.70)	0.003
Diabetes (%)	275 (19.50)	195 (11.20)	<0.001	170 (9.90)	300 (20.90)	<0.001
MetS (%)	508 (36.10)	351 (20.20)	<0.001	231 (13.50)	628 (43.70)	<0.001
CVD (%)	49 (3.50)	94 (5.40)	0.01	80 (4.70)	63 (4.40)	0.38
Glucose-Lowering agents' user (%)	89 (6.60)	94 (5.60)	0.15	72 (4.30)	111 (8.10)	<0.001
Lipid-lowering agents' user (%)	201 (14.40)	172 (10.00)	<0.001	175 (10.30)	198 (13.90)	0.001
Anti-hypertensive agents' user (%)	299 (21.20)	288 (16.50)	<0.001	282 (9.00)	305 (21.20)	<0.001
Family histort of diabetes (%)	544 (38.60)	595 (34.20)	0.005	594 (34.70)	545 (37.90)	0.03
Family histort of CDVs (%)	107 (7.60)	117 (6.70)	0.18	122 (7.10)	102 (7.10)	0.52
Family histort of HPTN (%)	753 (53.50)	665 (38.20)	<0.001	690 (40.30)	728 (50.70)	<0.001
Residual areas			<0.001			0.41
Rural	505 (35.90)	883 (50.70)		751 (43.90)	637 (44.30)	
Urban	903 (64.10)	858 (49.30)		961 (56.10)	800 (55.70)	
Physical activity quartiles			<0.001			0.16
Very low	368 (26.30)	392 (22.60)		393 (23.00)	367 (25.70)	
Low	224 (16.00)	208 (12.00)		225 (13.20)	207 (14.50)	
Moderate	508 (36.30)	624 (36.00)		629 (36.90)	503 (35.20)	
High	300 (21.40)	511 (29.50)		458 (26.90)	353 (24.70)	

*NAFLD, non-alcoholic fatty liver disease; PA, physical activity; BMI, body mass index; ALT, alanine transaminase; AST, aspartate transaminase; GGT, gamma-glutamyl transferase; ALKP, alkaline phosphatase; DAL, dietary acid load; NAE, net acid excretion; DED, dietary energy density; MetS, metabolic syndrome; CVD, cardiovascular disease; HPTN, hypertension*.

*Significant at p < 05 for Independent t-test for continuous variables and chi-square test for dichotomous variables*.

Non-alcoholic fatty liver disease subjects had a higher mean age, elevated liver enzymes (alanine transaminase, aspartate transaminase, gamma-glutamyl transferase, and alkaline phosphatase), lipid profiles (triglyceride, total cholesterol, HDL, and LDL), FBS, systolic and diastolic blood pressure, and DAL, as well as greater adiposity (body mass index and waist circumference), the prevalence of diabetes, hypertension, and family history of diabetes and hypertension (*p* < 0.001). In terms of other variables, there was no statistical difference between NAFLD and non-NAFLD subjects (*p* < 0.05).

Dietary energy and nutrient intakes of participants across different quartiles of major dietary patterns by gender are summarized in Supplementary Table 1. In all four quartiles of healthy dietary patterns, male participants had significantly different intakes of carbohydrate, protein, fat, cholesterol, polyunsaturated fatty acids, oleic acid, linoleic acid, eicosapentaenoic acid, docosahexaenoic acid, vitamin C, vitamin E, galactose, zinc, and Fe intake. Further, men in the different quartiles of the western pattern had significantly different intakes of carbohydrate, protein, cholesterol, monounsaturated fatty acids, linoleic acid, eicosapentaenoic acid, docosahexaenoic acid, total dietary fiber, vitamin A, glucose, fructose, sucrose, zinc, copper, and iron intake. Furthermore, a comparison of nutrient intakes in the traditional dietary pattern categories among men revealed a significant difference in terms of fat, cholesterol, saturated fatty acid, monounsaturated fatty acids, eicosapentaenoic acid, docosahexaenoic acid, vitamin A, vitamin C, vitamin E, galactose, and copper intake. An approximately similar pattern was also observed among women, which is presented in Supplementary Table 1.

The comparison of demographic, lifestyle factors, anthropometric, and biochemical assessments in the different categories of dietary patterns by sex are presented in Supplementary Table 2. The distribution of male participants in terms of DBP, FBS, and DAL was significantly different across healthy dietary pattern categories. In addition, there was a significant difference in triglyceride and DAL in the western dietary pattern categories. Further, there were significant differences in SBP, DBP, and gamma-glutamyl transferase across the traditional dietary pattern quartiles.

The female participants, on the other hand, had significantly different body mass index, waist circumference, triglyceride, HDL, SBP, and DAL distributions across the healthy dietary pattern categories. Moreover, there were no significant differences across different categories of the western dietary pattern according to participants' main characteristics. Furthermore, body mass index, waist circumference, SBP, FBS, and DAL significantly differed across the mixed dietary pattern.

### Identified Dietary Patterns

Factor analysis identified three dietary patterns. Table 2 shows the factor loading matrix of dietary patterns extracted by factor analysis. Eigenvalues, the scree plot test (Figure 1), and interpretability were evaluated to explain the food items.

**Figure 2:**
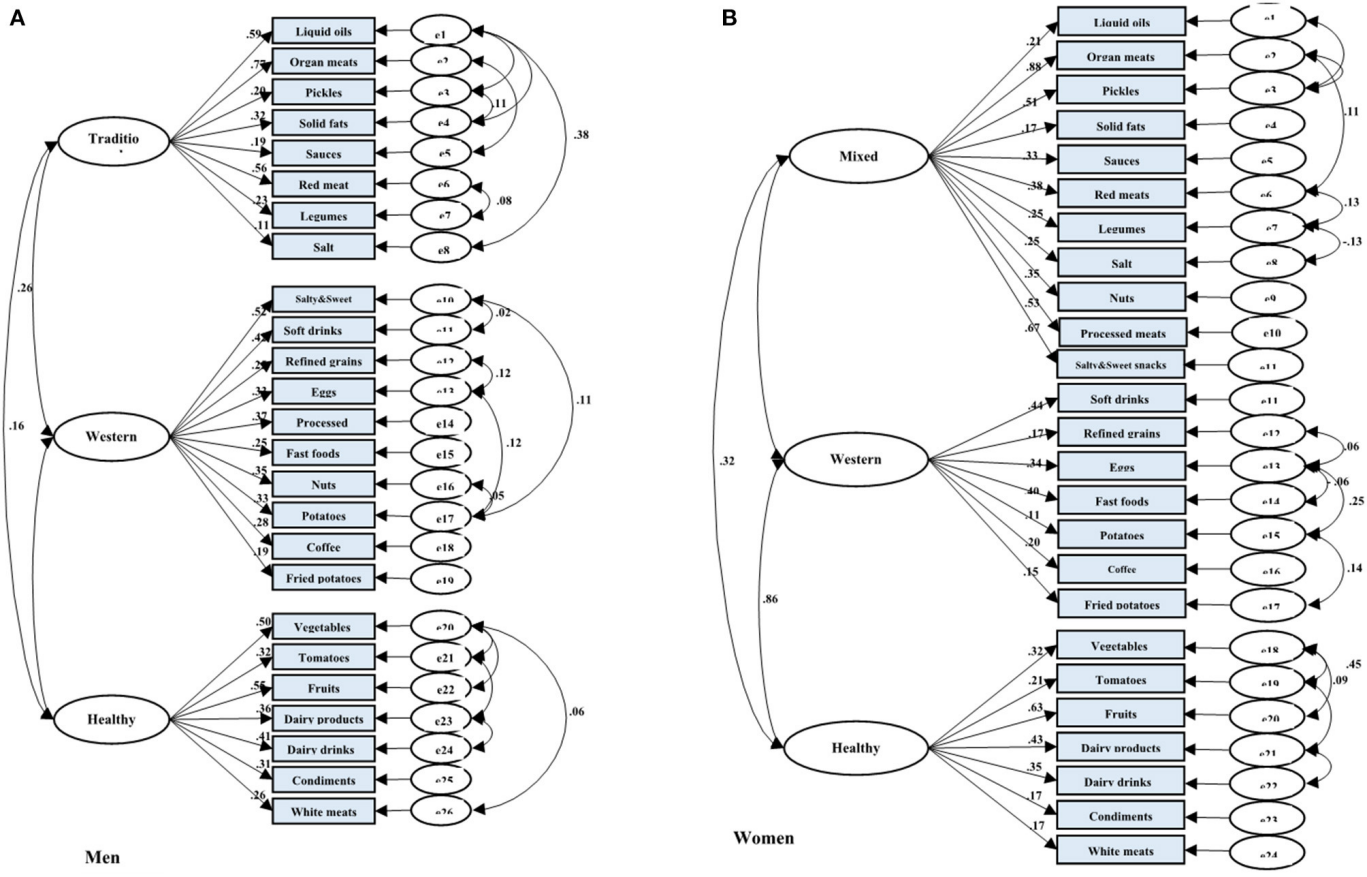
Results of the Confirmatory Factor Analysis (CFA) of the latent variables of dietary patterns and their indicator variables (food groups) among adults from AmolCS, Iran; **(A)** Men and **(B)** Women. Fit indices of measurement model of dietary patterns in **(A)** men: GFI = 0.94, AGFI = 0.92, SRMR = 0.05, RMSEA = 0.05, and in **(B)** women: GFI = 0.92, AGFI = 0.89, SRMR = 0.06, RMSEA = 0.05. Boxes indicate observed variables, and ellipses are latent variables in the model. All factor loadings and regression coefficients in the figure are standardized and have a *p* < 0.05.

Among men, three dietary patterns, including “traditional dietary pattern,” “healthy dietary pattern,” and “western dietary pattern” were established. The explained variances with traditional pattern (*eigenvalue* = 2.94), healthy pattern (*eigenvalue* = 2.39), and western pattern (*eigenvalue* = 1.61) were 9.47, 17.95, and 25.74, respectively. Subsequently, we put food groupings in three dietary patterns with higher factor loading into the confirmatory factor analysis model (Figure 2A). Finally, the traditional dietary pattern was loaded heavily on liquid oils, organ meats, pickles, solid fats, sauces, red meats, legumes, and salt; the healthy dietary pattern was also loaded heavily on vegetables, tomatoes, fruits, dairy drinks, dairy products, condiments, and white meats, and the western dietary pattern was also loaded heavily on salty and sweet snacks, soft drinks, processed meats, refined grains, eggs, nuts, fast foods, cooked and fried potatoes, and coffee.

Similarly, among women, three dietary patterns were detected by factor analysis. After considering the scree plot, factor loadings, and interpretability, the three dietary patterns, including “mixed dietary pattern,” “healthy dietary pattern,” and “unhealthy dietary pattern” were obtained by women. The explained variances with mixed pattern (*eigenvalue* = 3.46), healthy pattern (*eigenvalue* = 2.37), and unhealthy dietary pattern (*eigenvalue* = 1.54) were 11.99, 19.68, and 27.35, respectively. CFA was performed to confirm food grouping in dietary patterns (Figure 2B). Eventually, the mixed dietary pattern was loaded heavily in organ meats, pickles, nuts, processed meats, liquid oils, salty and sweet snacks, red meats, sauces, salt, legumes, and solid fats. The healthy dietary pattern was loaded heavily on vegetables, tomatoes, fruits, dairy products, dairy drinks, condiments, and white meats. Finally, the western dietary pattern was loaded heavily on soft drinks, fast foods, coffee, refined grains, fried potatoes, eggs, and cooked potatoes.

### Confirmatory Factor Analysis

The result of CFA indicated acceptable variance explanation and good data fit of the dietary patterns (in men: *GFI* = 0.94, *AGFI* = 0.92, *RMSEA* = 0.05, *SRMR* = 0.05; in women: *GFI* = 0.92, *AGFI* = 0.89, *RMSEA* = 0.05, *SRMR* = 0.06). All components were significantly related to the constructs of dietary pattern (*p* < 0.001; Figures 2A,B).

### Dietary Pattern and NAFLD

Crude and multiple adjusted *OR* and 95% *CI*s from multivariable logistic regression models, were used to analyze the association between major dietary patterns and NAFLD (Table 4). The first quartile (the lowest category of adherence to dietary patterns) was used in all models as the reference category. Full adjustment for potential confounders (model 3), in men only, revealed a significant positive relationship between affected with NAFLD and adherence to the western dietary pattern (Q2, Q3, Q4, *OR* = 1.16, 1.34, 1.39; 95% *CI* = 0.83–1.61, 0.96–1.85, 0.98–1.96, *p*_*trend*_ = 0.04, respectively). Nonetheless, adherence to other dietary patterns was not associated with odds of NAFLD in both genders.

**Table 4:** Odds ratios (95% CI) for NAFLD across Quartiles (Q) of dietary pattern scores.

**Dietary patterns**	**Q1 (low adherence)**	**Q2**	**Q4**	**Q4 (high adherence)**	***P*_*trend*_**
**Men (1,741)**					
Median score	−0.89	−0.41	0.10	1.10	
NAFLD subjects	195 (47.3)	192 (43.8)	200 (44.8)	213 (47.9)	
**Healthy pattern**					
Model 1	Ref	0.86 (CI:0.66–1.14)	0.90 (CI:0.69–1.18)	1.02 (CI:0.78–1.33)	0.64
Model 2	Ref	0.67 (CI:0.48–0.93)	0.73 (CI:0.52–0.73)	0.78 (CI:0.55–1.12)	0.42
Model 3	Ref	0.67 (CI:0.48–0.93)	0.73 (CI:0.52–1.04)	0.77 (CI:0.54–1.11)	0.40
**Western pattern**					
Median score	−0.86	−0.40	0.06	0.96	
NAFLD subjects	191 (42.9)	195 (43.7)	220 (49.3)	194 (48.0)	
Model 1	Ref	1.03 (CI:0.79–1.34)	1.28 (CI:0.98–1.67)	1.21 (CI:0.92–1.60)	0.11
Model 2	Ref	1.16 (CI:0.83–1.61)	1.33 (CI:0.96–1.84)	1.38 (CI:0.97–1.94)	0.05
Model 3	Ref	1.16 (CI:0.83–1.61)	1.34 (CI:0.96–1.85)	1.39 (CI:0.98–1.96)	0.047
**Traditional pattern**					
Median score	−0.51	−0.29	−0.08	0.38	
NAFLD subjects	206 (46.5)	187 (42.3)	195 (44.4)	212 (50.8)	
Model 1	Ref	0.83 (CI:0.64–1.10)	0.92 (CI:0.70–1.20)	1.18 (CI:0.90–1.55)	0.08
Model 2	Ref	0.94 (CI:0.62–1.07)	0.89 (CI:0.68–1.18)	1.11 (CI:0.79–1.41)	0.52
Model 3	Ref	0.91 (CI:0.65–1.26)	0.87 (CI:0.63–1.21)	1.06 (CI:0.76–1.54)	0.58
**Wemen (1,408)**					
Median score	−0.96	−0.38	0.13	1.06	
NAFLD subjects	145 (43.8)	165 (46.0)	161 (44.8)	166 (46.2)	
**Healthy pattern**					
Model 1	Ref	1.09 (CI:0.80–1.49)	1.04 (CI:0.76–1.41)	1.15 (CI:0.85–1.57)	0.42
Model 2	Ref	1.03 (CI:0.72–1.47)	1.00 (CI:0.69–1.45)	1.05 (CI:0.72–1.53)	0.82
Model 3	Ref	0.99 (CI:0.69–1.42)	1.00 (CI:0.69–1.45)	1.04 (CI:0.71–1.52)	0.87
Median score	−0.78	−0.32	0.07	0.85	
NAFLD subjects	189 (52.8)	167 (46.4)	153 (42.7)	128 (38.6)	
**Western pattern**					
Model 1	Ref	0.92 (CI:0.68–1.25)	0.91 (CI:0.67–1.25)	0.83 (CI:0.59–1.14)	0.46
Model 2	Ref	0.99 (CI:0.69–1.42)	1.06 (CI:0.73–1.54)	1.00 (CI:0.67–1.51)	0.92
Model 3	Ref	0.95 (CI:0.69–1.31)	0.92 (CI:0.66–1.29)	0.80 (CI:0.56–1.49)	0.21
Median score	−0.55	−0.32	−0.08	0.46	
NAFLD subjects	186 (52.2)	172 (47.9)	145 (40.3)	134 (40.2)	
**Mixed pattern**					
Model 1	Ref	0.89 (CI:0.66–1.21)	0.72 (CI:0.53–0.97)	0.80 (CI:0.58–1.10)	0.14
Model 2	Ref	0.99 (CI:0.69–1.42)	0.99 (CI:0.70–1.43)	1.09 (CI:0.75–1.61)	0.66
Model 3	Ref	1.03 (CI:0.72–1.47)	0.96 (CI:0.67–1.39)	0.98 (CI:0.67–1.46)	0.83

*Model 1: adjusted for demographic factor (age)*.

*Model 2: adjusted for demographic and lifestyle factors (age, smoking, waist circumstance, physical activity, energy intake, lowering serum lipid drugs, lowering HPTN drugs, lowering serum glucose drugs)*.

*Model 3: additional adjusted for residual areas, family history of hypertension, CVDs, and diabetes*.

### Structure Equation Modeling

**Figure 3:**
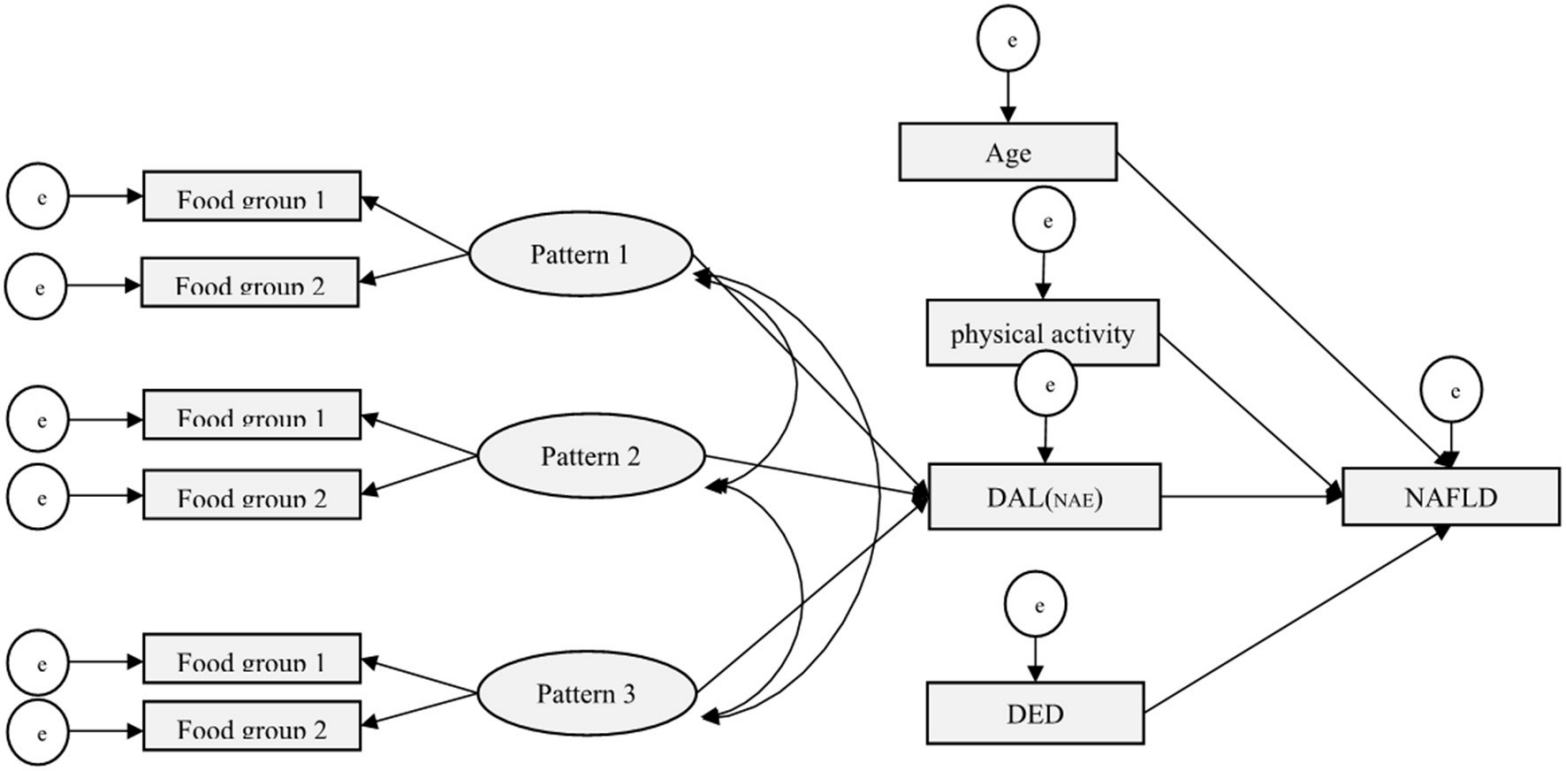
Conceptual SEM model for the association of demographic-behaviors factors, dietary patterns, and DAL with NAFLD.

**Figure 4:**
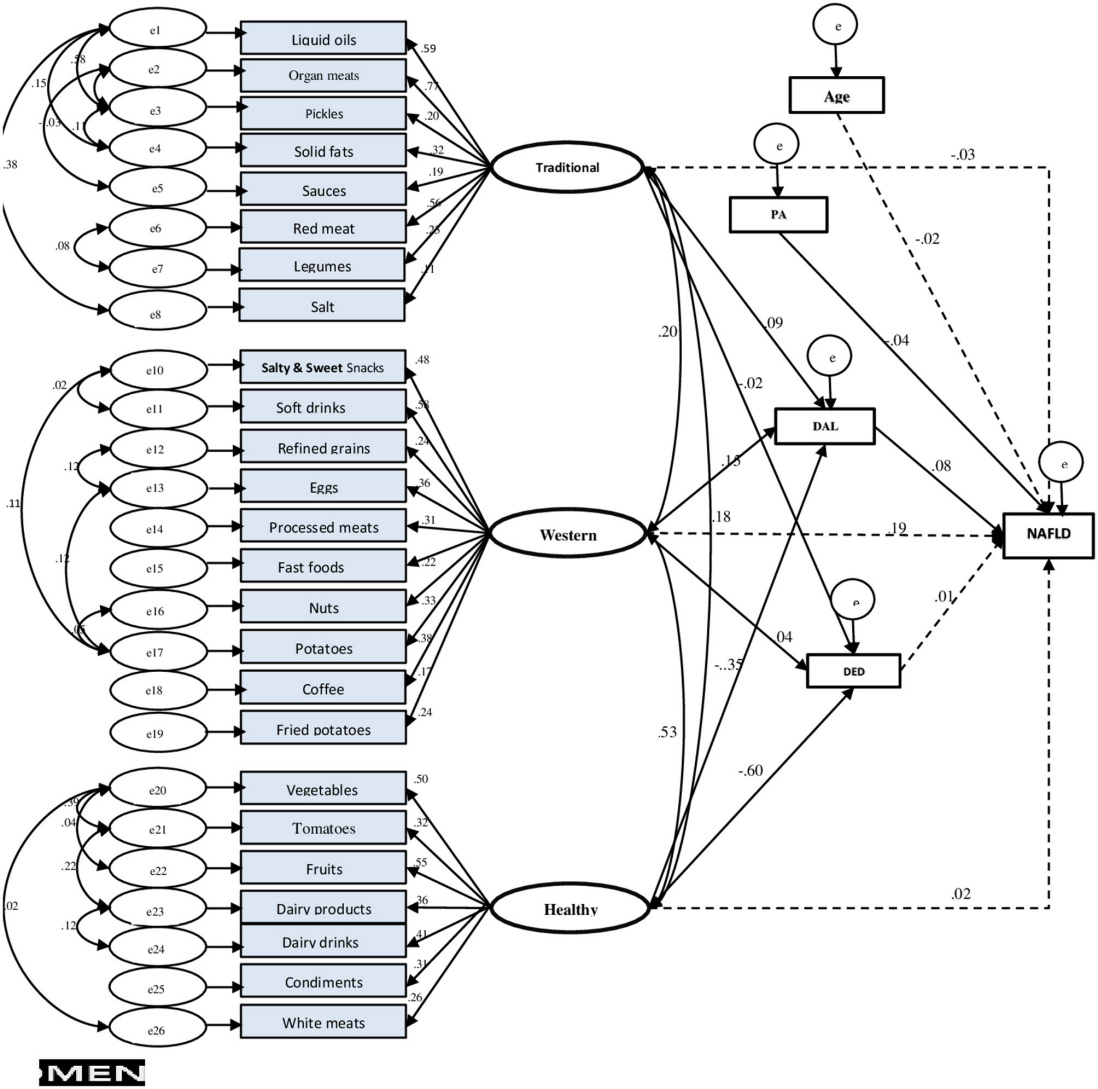
Final structural equation model (SEM) in men. Fit indices of SEM model: GFI = 0.94, AGFI = 0.93, SRMR = 0.04, RMSEA = 0.03. NAFLD, non-alcoholic fatty liver disease; PA, physical activity; DED, dietary energy density; DAL, dietary acid load.

Figure 3 shows the primary hypothesis model of the relationship between demographic-behaviors factors, dietary patterns, and DAL with NAFLD. Standardized regression weights (β) from the SEM are shown in Figure 4 to evaluate the conceptually derived model.

Only in men, dietary patterns had an association with NAFLD, and the GFI of the final model indicated an acceptable fit (*GFI* = 0.94, *AGFI* = 0.93, *SRMR* = 0.04, *RMSEA* = 0.03). Among potential mediator variables, including general and abdominal obesity, only the final DAL model fitted the data best. Adherence to the healthy dietary pattern, indirectly through DAL (β_*std*_ = −0.35, *p* < 0.006), decreased the NAFLD risk, while, concurrently, it had a direct negative impact on dietary energy density (DED) (β_*std*_ = −0.60, *p* < 0.001). The western dietary pattern indirectly (mediated by DAL, β_*std*_ = 0.15, *p* = 0.009) affected NAFLD risk. Moreover, the association between the traditional dietary pattern and NAFLD was found partly mediated by DAL (of dietary pattern to DAL, β_*std*_ = 0.09, *p* = 0.001), thus confirming our initial hypothesis.

## Discussion

In the current study, three distinct dietary patterns, including western, healthy, and traditional/mixed dietary, were identified using EFA and CFA in Iranian adults.

The western dietary pattern featured male participants with soft drinks, salty and sweet snacks, processed meats, refined grains, fast foods, eggs, cooked and fried potatoes, nuts, and coffee. Although in women, the western diet consisted of fast foods, soft drinks, refined grains, cooked and fried potatoes, coffee, and eggs. The healthy dietary pattern was characterized by vegetables, tomatoes, fruits, dairy drinks, dairy products, condiments, and white meats in both the genders. Accordingly, the food components of the PCA-derived western and healthy dietary patterns loaded in the current study were consistent with those identified by previous studies across different Iranian populations (40–42).

Also, the traditional pattern was highlighted by organ meats, liquid oils, pickles, solid fats, sauces, red meats, legumes, and salt. Indeed, this dietary pattern among women additionally included unhealthy food items such as processed meats and salty and sweet snacks, which was labeled as the mixed pattern. This pattern was very similar to western dietary patterns introduced in previous studies in Iran (43–45) and other nations (16, 46). In a recent study investigating the secular trend of dietary patterns among the Iranian population, from 2006 to 2017, Aghayan et al. reported that many western-style foods had been shifted to the traditional dietary pattern (41).

Our findings demonstrated that men who adhere to the western dietary pattern were more affected with NAFLD risk. This finding was consistent with previous studies conducted by Salehi-sahlabadi et al. (13) and Zelber-Sagi et al. (47). They demonstrated that the participants in the highest level of the western dietary pattern had the highest risk of NAFLD compared to the lowest level of western dietary pattern in the adult population. In the present study, the western dietary pattern was characterized by high loading on salty-sweet snacks, soft drinks, and refined grains which provided greater amounts of carbohydrate, cholesterol, sucrose, and lower amounts of protein, monounsaturated fatty acids, eicosapentaenoic acid, docosahexaenoic acid, total dietary fiber, zinc, copper, and iron, as well as triglyceride and DAL across higher quartiles of dietary pattern. Empirical evidence has confirmed the adverse metabolic effects of western and unhealthy dietary patterns in national (48) and international (49) studies. Previous studies highlighted that the higher consumption of soft drinks (50) and refined grains (51, 52) were associated with a greater risk of NAFLD. The mechanism behind this may be related to the provision of excess calorie intake and a large amount of sugar, such as sucrose and fructose (53). An experimental study demonstrated that consumption of both sucrose and fructose increased detrimental changes in hepatic lipid content and insulin resistance (54). Furthermore, other studies have reported that adherence to the western dietary pattern may be associated with insulin resistance, post-meal fat metabolism, and progression of NAFLD (7, 55). On the other hand, van Trijp revealed that replacing whole grains with refined grains yielded favorable changes in fecal microbiota composition and functionality, as well as improved liver health parameters (52).

In our study, we did not observe an independent association of traditional/mixed and healthy dietary patterns to NAFLD both in men and women. The possible explanation for this discrepancy could be that dietary patterns vary between ethnicities, cultural groups, and gender, as well as dietary patterns, which may change over time due to personal preferences and availability of food (56).

Using SEM, we evaluated the full mediate effects *via* DAL proxy of three mentioned dietary patterns on NAFLD. Although an indirect pathway between dietary patterns and potential adverse health effects such as obesity has been mentioned in the literature (57), empirical studies are limited on this subject. Ghaemi et al. (58), in a cross-sectional study conducted among 1,500 individuals referred to a nutrition clinic, reported the putative role of waist circumference as a mediator in the relationship between unhealthy and healthy dietary patterns and NAFLD.

According to Baron and Kenny (59), a full mediating effect occurs when the inclusion of a mediator reduces the strength of the relationship between independent and outcome variables to zero (e.g., non-significant). In our study, adjustment for DAL proxy weakened the relationship between dietary patterns and NAFLD, confirming our hypothesis that the association between dietary patterns and NAFLD would be mediated *via* DAL proxy.

Compared with healthy/prudent dietary patterns, acidic diets like, Western dietary patterns commonly contain a high consumption of animal-based diet and sugar-containing beverages and a low intake of plant-based diet such as vegetable, fruit, and whole grains (7). Alferink et al. concluded that an animal-based diet was positively correlated with all DAL proxies (e.g., PRAL, NEAP, and NAE) (60). Several population-based cohort studies found that animal proteins were independently associated with a higher prevalence of NAFLD (60–62).

Possible mechanisms to interpret these results included that acidogenic diets were related to increasing magnesium excretion and may have contributed to decreased insulin sensitivity (63). On the other hand, sulfur-containing amino acids (e.g., methionine and cysteine) found in animal-based proteins can be metabolized to sulfate after oxidation. This metabolic acidosis has been associated with various metabolic disorders (64). Moreover, long-term metabolic acidosis may increase adrenal cortisol production and subsequently lead to abdominal obesity and insulin resistance (65, 66).

Although previous epidemiologic studies have reported the association between NAFLD and dietary patterns in adults (11, 13, 14, 67–70), their simultaneous effects as a model were not discussed. The current study is the first study that provides greater understanding of how this association is mediated within an integrative model, and investigates the indirect effects of dietary patterns (through DAL proxy) on NAFLD.

The other strength of the present study is using a validated semi-quantitative FFQ developed for the Iranian population, which results in a better representation of the participants' dietary habits. In addition, this study includes a large number of participants, which makes the data and results robust. However, despite the novelty, this study does have some limitations. The cross-sectional design, in fact, precludes any causal inferences from being drawn. In the current study, we investigated dietary patterns using food intake data only, while other eating behaviors such as meal and snack patterns and cooking methods in the dietary pattern analysis are also suggested.

Further, the data of dietary, physical activity, and smoking habits were collected using self-reported questionnaires, where subjects' bias could have influenced the findings. Furthermore, the effect of unmeasured confounding factors cannot be excluded entirely. Nevertheless, to the best of our knowledge, our study is the first to investigate the indirect effects (through DAL proxy) of dietary patterns on NAFLD.

## Conclusion

A western dietary pattern comprising frequent intake of salty and sweet snacks, soft drinks, refined grains, processed meats, cooked and fried potatoes, eggs, and coffee was associated with a higher odds of NAFLD in an Iranian male population. Additionally, our findings might contribute to a mechanistic explanation for the association between dietary patterns and NAFLD *via* DAL proxy. However, further prospective studies with adequate consideration for acid-base biomarkers are needed.

## Data Availability Statement

The raw data supporting the conclusions of this article will be made available by the authors, without undue reservation.

## Ethics Statement

The studies involving human participants were reviewed and approved by Iran University of Medical Sciences (IUMS) Ethical Committee (No. IR.IUMS.REC.1399.1393). The patients/participants provided their written informed consent to participate in this study.

## Author Contributions

FZ, EP, ES, MN, AF, MRM, and AD were responsible for the study concept and design. AD and NM had full access to all data and took responsibility for the integrity of the data and the accuracy of the data analysis. MM, BA, HE, and MK were involved in data collection. AD, NM, CC, and EP analyzed and interpreted the data. MK and HE wrote the initial draft of the manuscript. FZ is the guarantor and takes responsibility for the article as a whole. All authors revised the manuscript critically for important intellectual content and approved the final manuscript.

## Funding

This research was conducted by a grant from the Gastrointestinal and Liver Diseases Research Center (GILDRC) and Iran University of Medical Sciences (IUMS) (Grant No. 99-2-30-19054).

## Conflict of Interest

The authors declare that the research was conducted in the absence of any commercial or financial relationships that could be construed as a potential conflict of interest.

## Publisher's Note

All claims expressed in this article are solely those of the authors and do not necessarily represent those of their affiliated organizations, or those of the publisher, the editors and the reviewers. Any product that may be evaluated in this article, or claim that may be made by its manufacturer, is not guaranteed or endorsed by the publisher.
