# Supplementary material for: Empirically-Derived Dietary Patterns in Relation to Non-Alcoholic Fatty Liver Diseases Among Adult Participants in Amol, Northern Iran: A Structural Equation Modeling Approach
Source: Front Nutr. 2022 Mar 28;9:821544. doi: 10.3389/fnut.2022.821544 (PMC8995896; doi:10.3389/fnut.2022.821544)
Supplement: Supplementary file 1 [file Table_1.DOCX]

**Supplementary Table 1. Adjusted means (SD)^a^ for dietary intakes across quartiles (Q) of dietary pattern scores**

| **Dietary patterns quartiles** | **Healthy pattern** | | | | **P** | **Western pattern** | | | | **P** | **Traditional pattern** | | | | **P** |
| --- | --- | --- | --- | --- | --- | --- | --- | --- | --- | --- | --- | --- | --- | --- | --- |
|  | **Q1** (n=412) | **Q2**  (n=438) | **Q3**  (n=446) | **Q4**  (n=445) |  | **Q1**  (n=445) | **Q2**  (n=446) | **Q3**  (n=446) | **Q4** (n=404) |  | **Q1**  (n=443) | **Q1**  (n=442) | **Q3**  (n=439) | **Q4**  (n=417) |  |
| **Men** |  |  |  |  |  |  |  |  |  |  |  |  |  |  |  |
| Carbohydrate **(g/d)** | 336.05  (2.26) | 335.72  (2.23) | 334.52  (2,24) | 320.65  (2.33) | <0.001 | 325.73  (2.23) | 332.55  (2.24) | 334.89  (2.23) | 333.61  (2.36) | 0.01 | 328.60  (2.40) | 333.54  (2.26) | 335.50  (2.25) | 328.76  (2.29) | 0.07 |
| **Protein (g/d)** | 100.21  (1.27) | 102.53  (1.20) | 103.70  (1.19) | 108.15  (1.21) | <0.001 | 106.78  (1.19) | 105.32  (1.19) | 102.18  (1.18) | 100.28  (1.25) | 0.001 | 102.98  (1.21) | 102.12  (1.20) | 105.07  (1.20) | 104.77  (1.25) | 0.26 |
| **Fat (g/d)** | 93.36  (1.48) | 86.92  (1.40) | 86.70  (1.39) | 87.90  (1.42) | 0.004 | 91.13  (1.39) | 87.82  (1.39) | 86.83  (1.38) | 88.80  (1.46) | 0.15 | 85.16  (1.39) | 86.14  (1.38) | 86.40  (1.38) | 97.33  (1.44) | <0.001 |
| **Cholestrol** | 366.26  (12.14) | 335.63  (11.45) | 327.85  (11.39) | 360.52  (11.58) | 0.04 | 355.94  (11.31) | 329.75  (11.32) | 333.13  (11.29) | 372.57  (11.92) | 0.02 | 346.46  (11.46) | 323.78  (11.33) | 330.61  (11.36) | 390.47  (11.85) | <0.001 |
| **SFA (g/d)** | 33.06  (.45) | 32.27  (.45) | 31.78  (.45) | 31.64  (.47) | 0.13 | 31.83  (.44) | 32.48  (.45) | 31.79  (.44) | 32.73  (.47) | 0.36 | 30.97  (.48) | 32.02  (.45) | 32.33  (.45) | 33.36  (.45) | 0.006 |
| **MUFA (g/d)** | 28.27  (.49) | 28.98  (.48) | 29.08  (.49) | 33.61  (.51) | <0.001 | 31.16  (.49) | 29.71  (.49) | 29.19  (.49) | 29.64  (.52) | 0.03 | 32.33  (0.52) | 29.51  (0.49) | 28.75  (0.49) | 29.32  (0.50) | <0.001) |
| **PUFA (g/d)** | 15.44  (.28) | 16.30  (.28) | 16.68  (.28) | 18.36  (.29) | <0.001 | 16.17  (.28) | 16.58  (.28) | 17.02  (.28) | 16.94  (.30) | 0.14 | 17.12  (.30) | 16.65  (.29) | 16.46  (.28) | 16.48  (.29) | 0.41 |
| **Oleic acid (g/d)** | 22.95  (.34) | 23.84  (.33) | 23.88  (.34) | 25.54  (.35) | <0.001 | 23.47  (.34) | 24.03  (.34) | 23.99  (.33) | 24.70  (.35) | 0.10 | 24.35  (.36) | 24.15  (.34) | 23.83  (.34) | 23.82  (.34) | 0.68 |
| **Linoleic acid (g/d)** | 11.74  (.27) | 12.63  (.27) | 12.79  (.27) | 14.11  (.28) | <0.001 | 12.08  (.27) | 12.68  (.27) | 13.18  (.27) | 13.30  (.28) | 0.008 | 13.25  (.29) | 12.86  (.27) | 12.73  (.27) | 12.39  (.28) | 0.23 |
| **EPA (g/d)** | .02  (.003) | .01  (.003) | .01  (.003) | .03  (.003) | <0.001 | 0.03  (0.003) | 0.02)  (0.003) | 0.02  (0.003) | 0.01  (0.004) | 0.001 | .04  (.003) | .01  (.003) | .01  (.003) | .01  (.003) | <0.001 |
| **DHA (g/d)** | .06  (.01) | .05  (.01) | .05  (.01) | .13  (.01) | <0.001 | .11  (.01) | .06  (.01) | .06  (.01) | .06  (.01) | <0.001 | .13  (.01) | .05  (.01) | .05  (.01) | .06  (.01) | <0.001 |
| **Total dietary fiber(g/d)** | 33.63  (14.91) | 36.88  (14.45) | 42.94  (14.59) | 43.68  (15.80) | 0.08 | 40.48  (.55) | 39.23  (.55) | 39.56  (.55) | 38.23  (.58) | 0.04 | 39.76  (.56) | 39.40  (.55) | 39.70  (.55) | 38.71  (.58) | 0.55 |
| **Vit. A (RAE)** | 821.20  (33.30) | 781.36  (31.43) | 779.52  (31.24) | 864.94  (31.77) | 0.17 | 869.22  (30.97) | 760.63  (31.04) | 755.46  (30.92) | 866.72  (32.66) | 0.006 | 736.97  (31.09) | 729.61  (30.73) | 765.60  (30.82) | 1026.54  (32.12) | <0.001 |
| **Vit.C (mg/d)** | 123.94  (3.63) | 130.18  (3.43) | 142.02  (3.41) | 173.59  (3.47) | <0.001 | 140.83  (3.49) | 137.45  (3.49) | 143.59  (3.48) | 150.15  (3.68) | 0.08 | 142.21  (3.54) | 134.13  (3.50) | 146.39  (3.51) | 148.98  (3.66) | 0.02 |
| **Vit. E (mg/d)** | 11.19  (.21) | 11.79  (.21) | 11.84  (.21) | 13.44  (.22) | <0.001 | 12.49  (.21) | 11.81  (.21) | 12.08  (.21) | 11.78  (.22) | 0.07 | 12.89  (.22) | 11.78  (.21) | 11.65  (.21) | 11.92  (.21) | <0.001 |
| **Glucose (g/d)** | 16.84  (.33) | 17.07  (.32) | 17.08  (.32) | 17.44  (.34) | 0.66 | 16.08  (.34) | 16.25  (.32) | 17.41  (.32) | 18.58  (.33) | <0.001 | 17.40  (.32) | 17.04  (.32) | 16.79  (.32) | 17.20  (.34) | 0.59 |
| **Fructose (g/d)** | 21.23  (.39) | 20.76  (.39) | 20.55  (.39) | 20.64  (.42) | 0.62 | 19.24  (.42) | 19.95  (.40) | 21.41  (.39) | 22.46  (.40) | <0.001 | 20.54  (.40) | 20.96  (.40) | 20.90  (.40) | 20.80  (.41) | 0.88 |
| **Sucrose (g/d)** | 39.77  (1.83) | 35.74  (1.73) | 33.40  (1.72) | 36.24  (1.75) | 0.09 | 31.00  (1.71) | 36.42  (1.71) | 38.17  (1.70) | 39.59  (1.80) | 0.003 | 34.79  (1.74) | 33.52  (1.72) | 38.36  (1.72) | 38.35  (1.80) | 0.12 |
| **Galactose (g/d)** | 4.99  (.12) | 4.45  (.12) | 4.38  (.12) | 3.81  (.13) | <0.001 | 4.26  (.12) | 4.51  (.12) | 4.42  (.12) | 4.49  (.13) | 0.50 | 3.73  (.13) | 4.22  (.12) | 4.38  (.12) | 5.28  (.12) | <0.001 |
| **Zn (mg/d)** | 11.30  (4.64) | 12.44  (3.73) | 14.36  (4.40) | 15.21  (4.72) | 0.009 | 13.94  (.12) | 13.55  (.12) | 13.00  (.12) | 12.95  (.13) | <0.001 | 13.19  (.13) | 13.32  (.13) | 13.40  (.13) | 13.59  (.13) | 0.20 |
| **Cu (mg/d)** | 2.03  (.03) | 1.95  (.03) | 1.96  (.03) | 1.94  (.03) | 0.30 | 2.11  (.03) | 1.95  (.03) | 1.89  (.03) | 1.92  (.03) | <0.001 | 1.82  (.03) | 1.89  (.03) | 1.94  (.03) | 2.24  (.03) | <0.001 |
| **Fe (mg/d)** | 26.61  (.62) | 25.26  (.59) | 26.75  (.59) | 28.17  (.60) | 0.009 | 28.08  (.58) | 26.11  (.58) | 25.90  (.58) | 26.73  (.61) | 0.03 | 26.17  (.59) | 26.31  (.59) | 27.25  (.59) | 27.11  (.61) | 0.48 |
| **Dietary patterns quartiles** | **Healthy pattern** | | | | **P** | **Western pattern** | | | | **P** | **Mixed pattern** | | | | **P** |
|  | **Q1** (n=331) | **Q2**  (n=359) | **Q3**  (n=359) | **Q4**  (n=359) |  | **Q1**  (n=358) | **Q2**  (n=360) | **Q3**  (n=358) | **Q4**  (n=332) |  | **Q1**  (n=356**)** | **Q1**  (n=359) | **Q3**  (n=360) | **Q4**  (n=333) |  |
| **Women** |  |  |  |  |  |  |  |  |  |  |  |  |  |  |  |
| **CHO (g/d)** | 289.50  (2.28) | 284.64  (2.15) | 282.07  (2.15) | 280.22  (2.19) | 0.02 | 277.87  (2.21) | 283.99  (2.14) | 284.70  (2.15) | 289.86  (2.27) | 0.004 | 288.64  (2.18) | 284.97  (2.14) | 283.26  (2.14) | 278.79  (2.26) | 0.02 |
| **Pro (g/d)** | 88.71  (1.10) | 90.74  (1.04) | 92.82  (1.04) | 94.86  (1.06) | 0.001 | 95.40  (1.07) | 92.90  (1.03) | 90.93  (1.04) | 87.87  (1.10) | <0.001 | 95.22  (1.05) | 93.52  (1.03) | 89.54  (1.03) | 88.93  (1.09) | <0.001 |
| **Fat (g/d)** | 79.83  (.98) | 81.29  (.92) | 81.96  (.92) | 82.38  (.94) | 0.27 | 83.11  (.95) | 80.88  (.92) | 81.14  (.92) | 80.39  (.97) | 0.21 | 77.77  (.92) | 79.89  (.91) | 82.78  (.91) | 85.40  (.96) | <0.001 |
| **cholestrol** | 305.86  (7.48) | 291.78  (7.24) | 302.39  (7.29) | 303.39  (7.70) | 0.53 | 277.67  (8.06) | 291.81  (7.28) | 304.37  (6.84) | 322.25  (6.65) | 0.02 | 290.13  (7.33) | 294.29  (7.21) | 291.74  (7.20) | 328.98  (7.59) | <0.001 |
| **SFA (g/d)** | 28.11  (.45) | 30.02  (.43) | 30.68  (.43) | 31.37  (.43) | <0.001 | 29.54  (.44) | 29.97  (.43) | 31.24  (.43) | 29.54  (.46) | 0.01 | 30.13  (.44) | 30.05  (.43) | 30.62  (.43) | 29.49  (.45) | 0.35 |
| **MUFA (g/d)** | 26.05  (.41) | 26.86  (.40) | 27.83  (.40) | 29.48  (.42) | <0.001 | 29.01  (.42) | 27.29  (.40) | 26.90  (.41) | 26.84  (.43) | 0.001 | 27.29  (.43) | 27.44  (.41) | 27.37  (.41) | 27.96  (.41) | 0.67 |
| **PUFA (g/d)** | 14.23  (.26) | 15.29  (.26) | 16.24  (.26) | 16.40  (.27) | <0.001 | 15.69  (.27) | 15.42  (.26) | 15.07  (.26) | 15.96  (.28) | 0.12 | 15.66  (.28) | 15.53  (.26) | 15.77  (.26) | 15.16  (.27) | 0.42 |
| **Oleic acid (g/d)** | 21.10  (.32) | 22.12  (.31) | 23.29  (.31) | 23.10  (.33) | <0.001 | 22.40  (.33) | 22.31  (.32) | 22.18  (.32) | 22.70  (.34) | 0.73 | 22.13  (.34) | 22.54  (.32) | 22.67  (.32) | 22.20  (.32) | 0.59 |
| **Linoleic acid (g/d)** | 10.71  (.26) | 11.78  (.25) | 12.73  (.25) | 12.82  (.27) | <0.001 | 11.89  (.27) | 11.88  (.26) | 11.66  (.26) | 12.61  (.27) | 0.07 | 12.38  (.27) | 12.09  (.26) | 12.17  (.26) | 11.38  (.26) | <0.001 |
| **EPA (g/d)** | .01  (.00) | .01  (.00) | .01  (.00) | .01  (.00) | 0.61 | .01  (.00) | .01  (.00) | .01  (.00) | .01  (.00) | 0.65 | .02  (.00) | .01  (.00) | .01  (.00) | .01  (.00) | 0.19 |
| **DHA (g/d)** | .05  .01 | .04  .01 | .06  .01 | .04  .01 | 0.62 | .06  (.01) | .05  (.01) | .03  (.01) | .05  (.01) | 0.34 | .06  (.01) | .03  (.01) | .04  (.01) | .06  (.01) | 0.06 |
| **Total dietary fiber(g/d)** | 35.29  (.57) | 35.11  (.53) | 35.54  (.53). | 36.15  (.54) | 0.56 | 36.18  (.55) | 35.67  (.53) | 35.09  (.53) | 35.13  (.57) | 0.48 | 37.85  (.54) | 35.47  (.53) | 34.51  (.53) | 34.19  (.56) | <0.01 |
| **Vit. A (RAE)** | 601.78  (28.45) | 640.63  (26.83) | 719.75  (26.82) | 813.00  (27.28) | <0.001 | 730.25  (27.88) | 646.94  (26.99) | 686.02  (27.16) | 721.41  (28.70) | 0.11 | 649.33  (27.11) | 610.39  (26.66) | 681.04  (26.62) | 852.75  (28.07) | <0.001 |
| **Vit.C (mg/d)** | 100.61  (3.46) | 110.99  (3.26) | 127.90  (3.26) | 159.33  (3.32) | <0.001 | 113.78  (3.50) | 117.80  (3.39) | 126.22  (3.41) | 144.38  (3.61) | <0.001 | 127.41  (3.48) | 114.37  (3.42) | 124.95  (3.42) | 134.72  (3.61) | 0.001 |
| **Vit. E (mg/d)** | 10.62  (.18) | 10.92  (.18) | 11.12  (.18) | 11.89  (.19) | <0.001 | 11.61  (.19) | 10.91  (.18) | 10.90  (.18) | 11.07  (.19) | 0.03 | 11.07  (.19) | 10.95  (.18) | 10.91  (.18) | 11.55  (.19) | 0.06 |
| **Glucose (g/d)** | 15.75  0.31) | 14.43  (0.29) | 15.32  (0.28) | 14.85  (0.28) | <0.001 | 15.05  (.30) | 14.75  (.29) | 14.79  (.29) | 15.75  (.31) | 0.07 | 13.04  (.30) | 14.24  (.28) | 15.24  (.28) | 17.62  (.24) | <0.001 |
| **Fructose (g/d)** | 19.28  (.39) | 17.82  (.38) | 18.91  (.38) | 18.18  (.40) | 0.03 | 18.42  (.40) | 18.31  (.38) | 18.36  (.38) | 19.17  (.41) | 0.39 | 16.03  (.39) | 17.62  (.37) | 18.87  (.37) | 21.50  (.38) | <0.001 |
| **Sucrose (g/d)** | 22.81  (1.08) | 24.37  (1.02) | 24.21  (1.02) | 26.21  (1.03) | 0.17 | 20.22  (1.03) | 22.41  (1.00) | 25.22  (1.00) | 30.31  (1.06) | <0.001 | 20.67  (1.02) | 21.98  (1.00) | 25.80  (1.00) | 29.62  (1.05) | <0.001 |
| **Galactose (g/d)** | 4.57  (.13) | 4.01  (.13) | 3.90  (.13) | 3.58  (.14) | <0.001 | 3.54  (.13) | 3.80  (.13) | 4.41  (.13) | 4.37  (.14) | <0.001 | 3.28  (.14) | 3.86  (.13) | 4.27  (.13) | 4.63  (.13) | <0.001 |
| **Zn (mg/d)** | 11.50  (.10) | 11.62  (.10) | 11.78  (.10) | 11.92  (.10) | 0.03 | 12.09  (.10) | 11.86  (.09) | 11.66  (.09) | 11.18  (.10) | <0.001 | 12.14  (.10) | 11.86  (.09) | 11.40  (.09) | 11.41  (.10) | <0.001 |
| **Cu (mg/d)** | 1.68  (.02) | 1.65  (.02) | 1.64  (.02) | 1.77  (.02) | 0.003 | 1.83  (.02) | 1.68  (.02) | 1.62  (.02) | 1.61  (.02) | <0.001 | 1.69  (.02) | 1.66  (.02) | 1.69  (.02) | 1.69  (.02) | 0.85 |
| **Fe (mg/d)** | 21.98  (.63) | 22.77  (.59) | 24.25  (.59) | 25.02  (.60) | 0.003 | 22.88  (.61) | 24.58  (.59) | 22.87  (.60) | 23.84  (.63) | 0.12 | 24.05  (.60) | 22.76  (.59) | 23.83  (.59) | 23.53  (.63) | 0.45 |

**Notes**: ^a^Adjusted for energy obtained from analysis of covariance (ANCOVA), except for DED. ^b^ Significant difference between the first and last quartile, obtained from Bonferroni’s post hoc test. **Abbreviations**: MUFAs, monounsaturated fatty acids; PUFAs, polyunsaturated fatty acids; SFAs, saturated fatty acids; EPA, eicosapentaenoic acid; DHA, docosahexaenoic acid.

**Supplementary Table 2. Adjusted means (SD)^a^ for Characteristics of participants across quartiles (Q) of dietary pattern scores**

| **Dietary patterns**  **quartiles** | **Healthy pattern** | | | | **P** | **Western pattern** | | | | **P** | **Traditional pattern** | | | | **P** |
| --- | --- | --- | --- | --- | --- | --- | --- | --- | --- | --- | --- | --- | --- | --- | --- |
|  | **Q1** (n=412) | **Q2**  (n=438) | **Q3**  (n=446) | **Q4**  (N=445) |  | **Q1** (n=445) | **Q2**  (n=446) | **Q3**  (n=446) | **Q4**  (n=404) |  | **Q1**  (n=443) | **Q1**  (n=442) | **Q3**  (n=439) | **Q4**  (n=417) |  |
| **Men** |  |  |  |  |  |  |  |  |  |  |  |  |  |  |  |
| **BMI (kg/m^2^)** | 26.82  (.22) | 26.97  (.20) | 26.62  (.20) | 26.85  (.21) | 0.70 | 26.74  (.20) | 26.71  (.20) | 26.92  (.20) | 26.88  (.21) | 0.88 | 26.85  (.21) | 26.47  (.20) | 26.90  (.20) | 27.05  (.21) | 0.24 |
| **Waist circumference (cm)** | 89.01  (.53) | 90.37  (.50) | 89.72  (.50) | 89.04  (.51) | 0.32 | 90.26  (.49) | 89.67  (.49) | 89.80  (.49) | 89.09  (.52) | 0.45 | 89.72  (0.53) | 88.75  (0.51) | 90.13  (0.51) | 90.33  (0.52) | 0.25 |
| **TG (mg/dl)** | 132.78  (4.32) | 144.05  (4.33) | 143.43  (4.32) | 132.88  (4.56) | 0.10 | 135.11  (4.63) | 150.24  (4.37) | 132.79  (4.35) | 135.48  (4.42) | 0.01 | 143.46  (4.40) | 136.04  (4.35) | 137.06  (4.36) | 137.01  (4.55) | 0.61 |
| **Total Cholesterol(mg/dl)** | 181.77  (1.97) | 180.14  (1.86) | 175.13  (1.85) | 176.70  (1.88) | 0.06 | 178.24  (1.84) | 177.27  (1.84) | 179.02  (1.84) | 178.98  (1.94) | 0.90 | 178.19  (1.87) | 176.89  (1.85) | 178.62  (1.86) | 179.83  (1.94) | 0.75 |
| **HDL** | 42.11  (.58) | 41.13  (.54) | 42.16  (.54) | 41.69  (.55) | 0.51 | 42.36  (.54) | 40.86  (.54) | 41.53  (.54) | 42.38  (.57) | 0.14 | 40.80  (.55) | 41.53  (.54) | 42.06  (.54) | 42.75  (.56) | 0.09 |
| **LDL** | 100.17  (1.33) | 99.18  (1.26) | 98.65  (1.25) | 97.15  (1.27) | 0.44 | 97.41  (1.24) | 99.22  (1.24) | 100.00  (1.24) | 98.36  (1.31) | 0.49 | 98.56  (1.26) | 97.89  (1.25) | 99.93  (1.25) | 98.67  (1.30) | 0.71 |
| **SBP (mmHg)** | 116.59  (.92) | 116.14  (.86) | 114.13  (.86) | 116.82  (.87) | 0.11 | 116.55  (.85) | 114.95  (.85) | 116.93  (.85) | 115.12  (.90) | 0.26 | 114.87  (.87) | 114.45  (.86) | 117.14  (.86) | 117.24  (.90) | 0.04 |
| **DBP (mmHg)** | 73.28  (.57) | 72.50  (.54) | 71.09  (.53) | 72.96  (.54) | 0.02 | 72.05  (.53) | 71.88  (.53) | 73.11  (.53) | 72.75  (.56) | 0.32 | 71.85  (.54) | 72.08  (.53) | 72.05  (.54) | 73.87  (.56) | 0.03 |
| **FBS (mg/dl)** | 107.95  (1.41) | 104.76  (1.42) | 101.19  (1.41) | 101.59  (1.49) | 0.002 | 102.67  (1.52) | 105.56  (1.44) | 104.07  (1.43) | 103.33  (1.45) | 0.53 | 102.21  (1.44) | 104.06  (1.43) | 103.44  (1.43) | 106.12  (1.49) | 0.31 |
| **ALT (mg/dl)** | 27.52  (1.03) | 27.25  (.97) | 27.17  (.97) | 28.38  (.98) | 0.81 | 26.90  (.96) | 26.61  (.96) | 28.53  (.96) | 28.36  (1.01) | 0.38 | 27.40  (.98) | 27.15  (.97) | 27.61  (.97) | 28.21  (1.01) | 0.89 |
| **AST (mg/dl)** | 23.12  (.58) | 23.01  (.55) | 22.79  (.54) | 24.42  (.55) | 0.15 | 22.86  (.54) | 23.05  (.54) | 23.32  (.54) | 24.21  (.57) | 0.34 | 23.06  (.55) | 23.77  (.54) | 23.04  (.54) | 23.52  (.57) | 0.73 |
| **GGT (mg/dl)** | 29.35  (.97) | 29.58  (.92) | 29.37  (.91) | 29.81  (93) | 0.98 | 29.76  (.91) | 28.95  (.91) | 30.23  (.91) | 29.16  (.96) | 0.75 | 29.09  (.92) | 30.04  (.91) | 29.09  (.91) | 29.93  (.95) | 0.81 |
| **DAL (NAE)** | 46.45  (1.26) | 46.56  (1.19) | 41.75  (1.17) | 34.94  (1.22) | <0.001 | 38.35  (1.20) | 41.61  (1.18) | 43.06  (1.18) | 46.68  (1.20) | <0.001 | 43.36  (1.17) | 44.56  (1.18) | 42.14  (1.17) | 40.65  (1.21) | 0.05 |
| **Dietary patterns quartiles** | **Healthy pattern** | | | | **P** | **Western pattern** | | | | **P** | **Mixed pattern** | | | | **P** |
|  | **Q1** (n=331) | **Q2**  (n=359) | **Q3**  (n=359) | **Q4**  (n=359) |  | **Q1**  (n=358) | **Q2**  (n=360) | **Q3**  (n=358) | **Q4**  (n=332) |  | **Q1**  (n=356) | **Q1**  (n=359) | **Q3**  (n=360) | **Q4**  (n=333) |  |
| **Women** |  |  |  |  |  |  |  |  |  |  |  |  |  |  |  |
| **BMI (kg/m^2^)** | 30.13  (.28) | 30.12  (.28) | 29.23  (.28) | 28.80  (.29) | 0.002 | 29.19  (.29) | 30.04  (.28) | 29.29  (.28) | 29.80  (.28) | 0.10 | 30.44  (0.29) | 29.73  (0.27) | 29.07  (0.26) | 29.20  (0.26) | 0.003 |
| **Waist circumference (cm)** | 91.31  (.64) | 88.76  (.62) | 86.78  (.63) | 85.21  (.66) | <0.001 | 87.58  (.67) | 88.43  (.63) | 87.89  (.63) | 88.32  (.64) | 0.78 | 91.39  (.66) | 89.13  (.62) | 86.44  (.59) | 85.74  (.59) | <0.001 |
| **TG (mg/dl)** | 139.12  (4.94) | 132.76  (4.64) | 118.34  (4.47) | 125.08  (4.48) | 0.01 | 134.66  (4.83) | 131.72  (4.68) | 121.93  (4.71) | 125.28  (4.98) | 0.24 | 120.00  (4.98) | 128.82  (4.69) | 128.29  (4.69) | 136.06  (4.77) | 0.15 |
| **Total Cholesterol(mg/dl)** | 181.00  (2.35) | 182.40  (2.22) | 182.35  (2.22) | 188.69  (2.26) | 0.08 | 183.75  (2.28) | 187.85  (2.21) | 179.77  (2.22) | 183.22  (2.35) | 0.08 | 186.36  (2.34) | 184.18  (2.20) | 178.97  (2.12) | 185.50  (2.12) | 0.06 |
| **HDL** | 44.87  (.66) | 45.99  (.62) | 45.69  (.62) | 47.70  (.63) | 0.01 | 45.25  (.64) | 46.82  (.62) | 46.28  (.62) | 45.99  (.66) | 0.35 | 45.40  (.66) | 46.15  (.61) | 46.14  (.59) | 46.58  (.59) | 0.62 |
| **LDL** | 98.85  (1.50) | 99.09  (1.41) | 100.10  (1.41) | 100.73  (1.44) | 0.79 | 100.62  (1.45) | 102.10  (1.41) | 98.34  (1.42) | 97.62  (1.50) | 0.11 | 102.58  (1.49) | 100.36  (1.40) | 97.38  (1.35) | 98.88  (1.35) | 0.06 |
| **SBP (mmHg)** | 117.86  (1.09) | 113.82  (1.06) | 110.88  (1.07) | 111.24  (1.13) | <0.001 | 111.66  (1.14) | 113.42  (1.07) | 114.44  (1.07) | 114.32  (1.09) | 0.28 | 115.90  (1.13) | 114.17  (1.06) | 112.48  (1.02) | 111.74  (1.02) | 0.03 |
| **DBP (mmHg)** | 69.88  (.68) | 71.47  (64) | 70.69  (.64) | 71.18  (.65) | 0.35 | 71.05  (.66) | 71.31  (.64) | 70.37  (.64) | 70.54  (.68) | 0.72 | 71.23  (.68) | 71.08  (.63) | 70.50  (.61) | 70.54  (.61) | 0.80 |
| **FBS (mg/dl)** | 120.66  (2.16) | 107.41  (2.09) | 102.95  (2.10) | 103.46  (2.22) | <0.001 | 107.82  (2.26) | 107.32  (2.13) | 108.98  (2.13) | 110.65  (2.16) | 0.72 | 115.60  (2.23) | 108.02  (2.10) | 103.59  (2.02) | 108.43  (2.02) | 0.001 |
| **ALT (mg/dl)** | 20.06  (.78) | 19.46  (.74) | 20.48  (.74) | 19.20  (.75) | 0.60 | 20.76  (.76) | 19.29  (.73) | 19.66  (.74) | 19.46  (.78) | 0.52 | 20.26  (.78) | 20.04  (.73) | 19.54  (.70) | 19.41  (.70) | 0.82 |
| **AST (mg/dl)** | 19.69  (.46) | 19.40  (.43) | 19.76  (.43) | 19.11  (.44) | 0.72 | 20.40  (.45) | 19.70  (.43) | 19.04  (.43) | 18.74  (.46) | 0.05 | 19.67  (.46) | 20.11  (.43) | 19.44  (.41) | 18.77  (.41) | 0.14 |
| **GGT (mg/dl)** | 27.31  (1.01) | 23.55  (.98) | 23.38  (.98) | 22.19  (1.04) | 0.004 | 22.78  (1.04) | 23.87  (.98) | 24.85  (.98) | 24.97  (1.00) | 0.42 | 25.43  (1.04) | 23.93  (.97) | 24.03  (.94) | 23.33  (.94) | 0.51 |
| **DAL(NAE)** | 45.00  (1.19) | 41.36  (1.12) | 34.92  (1.12) | 27.90  (1.15) | <0.001 | 35.08  (1.19) | 41.91  (1.13) | 36.66  (1.17) | 35.15  (1.22) | 0.003 | 43.41  (1.17) | 36.63  (1.12) | 36.63  (1.12) | 30.49  (1.22) | <0.001 |

**Notes**: ^a^Adjusted for energy obtained from analysis of covariance (ANCOVA). ^b^ Significant difference between the first and last quartile obtained from Bonferroni’s post hoc test.
